# Functional Display of Platelet-Binding VWF Fragments on Filamentous Bacteriophage

**DOI:** 10.1371/journal.pone.0073518

**Published:** 2013-09-03

**Authors:** Andrew Yee, Fen-Lai Tan, David Ginsburg

**Affiliations:** 1 Life Sciences Institute, University of Michigan, Ann Arbor, Michigan, United States of America; 2 Departments of Internal Medicine, Human Genetics, and Pediatrics, University of Michigan, Ann Arbor, Michigan, United States of America; 3 Howard Hughes Medical Institute, University of Michigan, Ann Arbor, Michigan, United States of America; National Cerebral and Cardiovascular Center, Japan

## Abstract

von Willebrand factor (VWF) tethers platelets to sites of vascular injury via interaction with the platelet surface receptor, GPIb. To further define the VWF sequences required for VWF-platelet interaction, a phage library displaying random VWF protein fragments was screened against formalin-fixed platelets. After 3 rounds of affinity selection, DNA sequencing of platelet-bound clones identified VWF peptides mapping exclusively to the A1 domain. Aligning these sequences defined a minimal, overlapping segment spanning P1254–A1461, which encompasses the C1272–C1458 cystine loop. Analysis of phage carrying a mutated A1 segment (C1272/1458A) confirmed the requirement of the cystine loop for optimal binding. Four rounds of affinity maturation of a randomly mutagenized A1 phage library identified 10 and 14 unique mutants associated with enhanced platelet binding in the presence and absence of botrocetin, respectively, with 2 mutants (S1370G and I1372V) common to both conditions. These results demonstrate the utility of filamentous phage for studying VWF protein structure-function and identify a minimal, contiguous peptide that bind to formalin-fixed platelets, confirming the importance of the VWF A1 domain with no evidence for another independently platelet-binding segment within VWF. These findings also point to key structural elements within the A1 domain that regulate VWF-platelet adhesion.

## Introduction

von Willebrand factor (VWF) is a multimeric glycoprotein that is central to development of a hemostatic platelet plug. The A1 domain of VWF has been previously identified as the primary ligand for the platelet receptor, GPIb (reviewed in [1]). Transient tethering between the A1 domain of VWF and GPIb facilitates rapid platelet immobilization to sites of vascular injury. Crystal structures of the A1-GPIb complex show that GPIb forms a concave pocket with leucine-rich repeats that interface with the VWF A1 domain following conformational changes induced by biochemical cofactors or by mutations in the A1 domain associated with von Willebrand disease (VWD) type 2B [2], [3], [4]. In the circulation, hydrodynamic forces stretch VWF from a compacted to an extended shape, exposing the A1 domain to passing platelets. In diseased blood vessels where shear rates may exceed 10,000 s^−1^, conformational changes in the A1 domain of immobilized, extended VWF result in platelet adhesion via high affinity binding between A1 and GPIb [5], [6], [7].

The architecture in and around the A1 domain regulate VWF binding to platelets. The A1 domain of VWF contains a single intramolecular disulfide bond between C1272 and C1458 that may optimize its structure for platelet binding [8], [9]. The residues N-terminal to C1272 have been proposed to allosterically hinder binding between the A1 domain and GPIb [10], [11], [12]. The contribution of other VWF regions to GPIb binding has been less characterized.

Phage display is a powerful tool for studying protein interactions and provides an unbiased, comprehensive approach to interrogate all VWF residues involved in platelet binding. This method, which expresses large libraries of peptides or proteins (up to ∼10^9^ independent clones) on the surface of a bacteriophage, has been used for a variety of applications [13]. M13 filamentous phage infect f-pili-bearing *E. coli* and exploit the host’s cellular machinery to propagate phage particles without killing the bacterium. Typically, the phage genome is engineered to fuse a polypeptide or the variable region of single chain antibodies to the N-terminus of the minor coat protein, pIII. The fusion protein produced in the cytoplasm is transported into the periplasm where phage particles assemble at sites of cytoplasmic/periplasmic membrane fusions, encapsulating the phage DNA containing the cloned insert and thus, linking the DNA sequence to the protein it encodes. After affinity selection (“panning”), phage DNA (now enriched) are recovered by infecting naïve bacteria for amplification and subsequent phage particle production (“phage rescue”). This process is typically repeated for 3–4 additional cycles, with continued enrichment for the specific class of recombinant phage.

We previously constructed a random VWF fragment, filamentous phage library to map the epitopes for an anti-VWF antibody [14]. Here, we extend this approach to finely map the platelet-binding domain of VWF and to identify VWF fragments with enhanced affinity for platelets.

## Materials and Methods

### Phage Display Library and Vector Construction

Construction of a filamentous phage display wild type VWF (wtVWF) cDNA fragment library containing ∼7.7×10^6^ independent clones with VWF cDNA fragments ranging in size from ∼100 bp to ∼700 bp has been previously described [14]. The size of VWF cDNA fragments cloned into the phagemid allowed expression and display of peptide lengths (∼33 aa to ∼233 aa) sufficient to encompass the intramolecular C1272–C1458 cystine loop (187 aa) of the A1 domain. Because these cDNA fragments were randomly inserted between the C-terminus of the signaling sequence and the N-terminus of pIII, only 1 in 24 independent clones were expected to produce a properly displayed VWF peptide (1/2 with correct orientation, 1/3 in frame at the 5′ end, 1/3 in frame at the 3′ end, and 3/4 of the VWF plasmid contain VWF cDNA). Of the predicted ∼540,000 unique, VWF peptide fragments that range in size from ∼33 aa to ∼233 aa, ∼1128 span C1272 and C1458, the cysteines that form the single intramolecular disulfide bond of the A1 domain. Taking these considerations together, only ∼1∶11,500 of the ∼7.7×10^6^ independent clones (∼670) in the original VWF fragment library would be predicted to encode a VWF peptide fragment that includes the C1272–C1458 intramolecular disulfide bond.

To construct a phage display library of randomly mutagenized A1 domains (mtA1) with mutations possible from V1245 to P1465, the VWF cDNA encoding VWF Q1238 to P1471 was amplified by error-prone-PCR, as previously described [15], using primers (5′-TGGCCCAGCCGGCCCAGGAGCCGGGAGGCCTGGT-3′ and 5′-TGCGGCCGCGTGGGGGGGCAGAGTAGGAGG-3′) and subcloned into the phagemid vector, pCANTAB5e (GE Life Sciences), at the SfiI and NotI restriction sites. The ligation mix was electroporated into TG-1 cells (Agilent) and cultured on LB agar, Lennox (BD Diagnostic Systems) plates containing 100 µg/mL ampicillin and 2% glucose at 37°C overnight. All ampicillin-resistant clones were scraped into LB containing 20% glycerol and stored at −80°C.

**Figure 1:**
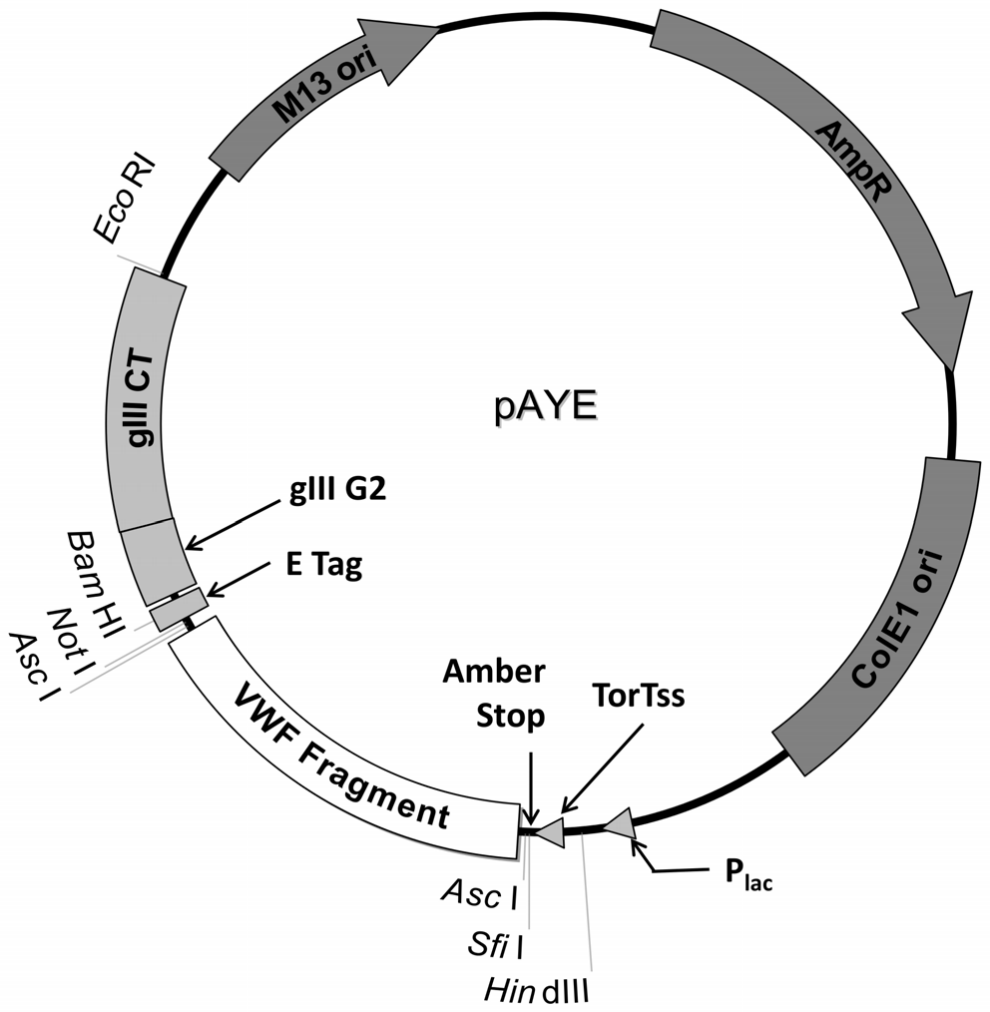
Phagemid pAYE. Expression of cloned VWF fragments is driven by the lac promoter (P_lac_). An amber stop placed between the TorT signaling sequence (TorTss) and the VWF fragment reduces the expression rate to prevent compartmentalization into inclusion bodies. The cloned VWF fragment is C-terminally fused to an E tag and a tandem, truncated M13 pIII coat protein composed of only a glycine-serine rich linker (gIII G2) and the pIII anchor domain (gIII CT). Relevant restriction sites are indicated. pAYE confers ampicillin resistance (AmpR) to host *E. coli* and contains a high copy number origin of replication (ColE1 ori) and a bacteriophage origin of replication (M13 ori). See Text S1 and Table S1 or GenBank (KF384455) for the complete pAYE sequence.

For competitive, single round panning experiments, the pAYE phagemid vector (Figure 1, Text S1, and Table S1) was constructed from pCANTAB5e using standard PCR and cloning techniques to 1) replace the gIII signaling sequence with the TorT signaling sequence, 2) move the amber stop from the 3′ end of the cloned cDNA to between the TorT signaling sequence and the 5′ end of the cloned cDNA, and 3) remove the infective domains (N1 and N2) of pIII. The DNA sequence for pAYE has been deposited in GenBank (KF384455). The TorT signaling sequence facilitates cotranslational-translocation and stable display of longer peptides (∼300 amino acids) [16]. The VWF cDNA encoding the A1 domain (Q1238–V1476) and the A3 domain (S1671–G1874) were PCR amplified and subcloned into pAYE at the AscI or AscI/NotI restriction sites, respectively, generating pAYE-A1^wt^ and pAYE-A3. The cysteines (C1272 and C1458) in pAYE-A1^wt^ were mutated to alanine by site-directed mutagenesis (Agilent) according to the manufacturer’s instructions, generating pAYE-A1^C1272/1458A^. The sequences of pAYE-A1^wt^, pAYE-A3, and pAYE-A1^C1272/1458A^ were verified by Sanger sequencing. pAYE-A1^wt^, pAYE-A3, and pAYE-A1^C1272/1458A^ were maintained in XL1-Blue MRF’ cells (Agilent) and stored at −80°C as 20% glycerol stocks.

### Platelet Panning

#### Library screens

Phage particles from the above libraries were generated using the expression module of the RPAS kit (GE Life Sciences) according to the manufacturer’s instruction. Formalin-fixed platelets (Bio/Data Corp.) were blocked by incubating in phosphate buffered saline (PBS) containing 2.5% (w/v) nonfat powdered milk. Freshly prepared phage particles (∼5×10^10^ phage forming units (pfu)) composed of either 100% wtVWF fragment library or 99% wtVWF fragment library +1% mtA1 library in PBS containing 2.5% (w/v) bovine serum albumin (BSA) were panned against ∼10^7^ blocked, formalin-fixed platelets in the presence or absence of 2 U/mL botrocetin (Centerchem) at 22°C for 30 min. Since botrocetin, unlike ristocetin, is insensitive to multimer size [17], we reasoned that botrocetin was also likely to be less sensitive to the absence of other contiguous VWF domains, as the crystal structure for the ternary complex [4] suggests, and thus more effective for screening a VWF peptide fragment library. Also, ristocetin, an antibiotic, would likely impair phagemid recovery (which requires growth in *E. coli*). Panned platelets were separated from unbound phage particles by centrifugation (10,000 g for 5 min) through a 20% sucrose cushion and subsequently washed with PBST (PBS +0.1% Tween-20). The wash stringency was increased for successive rounds of panning: round 1) twice at 4°C for 15 min, round 2) three times at 22°C for 15 min, rounds 3 and 4) three times at 22°C for 30 min then once at 4°C, overnight. Platelet-bound phage particles were recovered by incubation with log phase (OD_600 nm_ = 0.3–0.5) TG1 cells at room temperature for 15 min. Ampicillin resistance was developed by culturing the infected TG1 cells at 37°C for 45 min in LB. An aliquot of ampicillin-resistant TG1 cells was plated on LB-agar containing 100 µg/mL ampicillin and 2% glucose for colony screens. The remaining cells were infected with helper phage M13KO7 (GE Life Sciences), according to the manufacturer’s instructions, to rescue phage particles displaying selected VWF fragments for subsequent rounds of panning.

Colonies harboring recovered phagemid were further screened for platelet binding using the detection module of the RPAS kit (GE Life Sciences) according to the manufacturer’s instructions. Briefly, randomly selected colonies were individually cultured and infected with M13KO7 to rescue phage particles that were subsequently tested for platelet binding in an ELISA-based assay. 96-well plates were coated with monoclonal anti-human GPIbα (CD42b, Clone SZ2, Beckman Coulter) at 10 µg/ml in PBS and subsequently loaded with ∼2×10^7^ formalin-fixed platelets in PBS. The interaction between the antibody and GPIb at the surface of wells would not be expected to affect phage binding to GPIb that are situated within the well and away from the surface of the ELISA plate. After removing unbound platelets, wells were blocked with 2.5% nonfat milk/PBS. Individually rescued phage in 2.5% BSA/PBS were then incubated with the immobilized formalin-fixed platelets at 22°C for 30 min in the presence of 2 U/mL botrocetin. Bound phage were detected with HRP conjugated, monoclonal anti-M13 filamentous phage coat protein pVIII antibody as previously described [14]. The phagemid inserts from ELISA positive clones were PCR amplified and Sanger sequenced with primers (5′-CAACGTGAAAAAATTATTATTCGC-3′ and 5′-GTAAATGAATTTTCTGTATGAGG-3′).

#### Competitive, single round panning

XL1-Blue MRF’ cells harboring pAYE-A1^wt^, pAYE-A3, or pAYE-A1^C1272/1458A^ were cultured in 2×YT (10 mg/mL yeast extract +17 mg/mL tryptone +5 mg/mL NaCl) containing 100 µg/mL ampicillin +2% (w/v) glucose to log phase (OD_600 nm_ = 0.3–0.5) at 37°C and then infected with M13KO7 at a multiplicity of infection of ∼200 at 37°C for 1 hr. Infected cells were collected by centrifugation at 2,000 g for 15 min at room temperature, resuspended in 2×YT containing 100 µg/mL ampicillin +50 µg/mL kanamycin +0.1 mM IPTG and cultured at 30°C overnight. Phage particles were separated from *E. coli* by centrifugation (2,000 g, 4°C, 15 min) followed by sterile-filtration (0.2 µm filter). Phage preparations were concentrated by precipitation twice with 1/5^th^ volume of 20% polyethylene glycol-8000/2.5 M NaCl on ice for at least 30 min prior to centrifugation (16,000 g, 4°C, 30 min); the phage pellet was resuspended in sterile TBS (50 mM Tris-HCl, pH = 7.4; 150 mM NaCl) after the final centrifugation. To titrate phage stocks, XL1-Blue MRF’ cells were grown to mid-log phase, infected with serial dilutions of the resuspended phage pellet at 37°C for 1 hr, and plated on LB-agar containing 100 µg/mL ampicillin and 2% glucose.

In competitive, single round panning experiments, ∼2×10^7^ formalin-fixed platelets blocked in 2.5% BSA/TBS-T (TBS +0.05% Tween-20) were first incubated with A3 phage at room temperature for 1 hr and then panned against A1^wt^, A1^C1272/1458A^, or equal amounts of both in the presence or absence of 1 U/mL botrocetin for another hour at room temperature. The total input phage particles amounted to 4×10^11^ pfu in 400 µL composed of 95% A3+ balanced titrations of A1^wt^ or A1^C1272/1458A^. Panned platelets were separated from unbound phage particles by centrifugation at 16,000 g for 5 min and then washed 5 times with TBS-T and once with TBS. Phagemids were recovered by incubating washed platelets with XL1-Blue MRF’ cells (cultured to OD_600 nm_ = 0.3–0.5) at 37°C for 1 hr. Infected *E. coli* were plated on LB-agar containing 100 µg/mL ampicillin +2% (w/v) glucose for phage titrations and colony screens. Ampicillin-resistant colonies were randomly selected for PCR analysis using primers flanking the phagemid cloning site (5′-CCATGATTACGCCAAGCTTTGGAGCC-3′ and 5′- CGGCACCGGCGCACCTG-3′); A3 phage gave 770 bp PCR products while A1^wt^ and A1^C1272/1458A^ phage gave 884 bp PCR products. A1^wt^ and A1^C1272/1458A^ amplicons were distinguished by Sanger sequencing (primer = 5′- TTTGGAGCCTTTTTTTTGGAGATTTT-3′) or by restriction fragment length polymorphism (RFLP) analysis with PstI.

### Disulfide Structure Analysis

For periplasmic expression of VWF fragments, pAYE was converted into an expression plasmid (pAYEX) by subcloning an adaptor (5′- GATCCGCTGGAACCGCGTTAATAAG-3′ and 5′- AATTCTTATTAACGCGGTTCCAGCG-3′) at the BamHI and EcoRI sites to remove gIII and introduce a stop codon after the E tag. XL1-Blue MRF’ cells bearing pAYEX-A1^wt^, pAYEX- A1^C1272/1458A^, or pAYEX-A3 were cultured in 2×YT containing 100 µg/mL ampicillin +0.1 mM IPTG at 30°C, overnight. Periplasmic protein was 1) harvested by osmotic shock using PeriPreps (Epicentre), 2) quantified using the Coomassie protein assay kit (Pierce), 3) fractionated (2 µg total protein per sample) by electrophoresis in SDS-PAGE under reducing (20 mM DTT) or non-reducing conditions, and 4) transferred onto PVDF membranes (Bio-Rad) using native-gel transfer conditions (25 mM Tris +25 mM glycine, pH = 9.3; 20% methanol). The membrane was blocked with 5% nonfat milk/TBS-T, probed with 1∶30,000 goat-anti-E Tag-HRP (Abcam) in 5% nonfat milk/TBS-T, and developed with ECL (Thermo Scientific).

### Statistics

Significance was tested by calculating the probability from the hypergeomteric distribution or from the chi-square (χ^2^) distribution, where indicated.

## Results

### Phage Expressing VWF A1 Domain Peptide Fragments Exhibit Specific Platelet-binding

**Figure 2:**
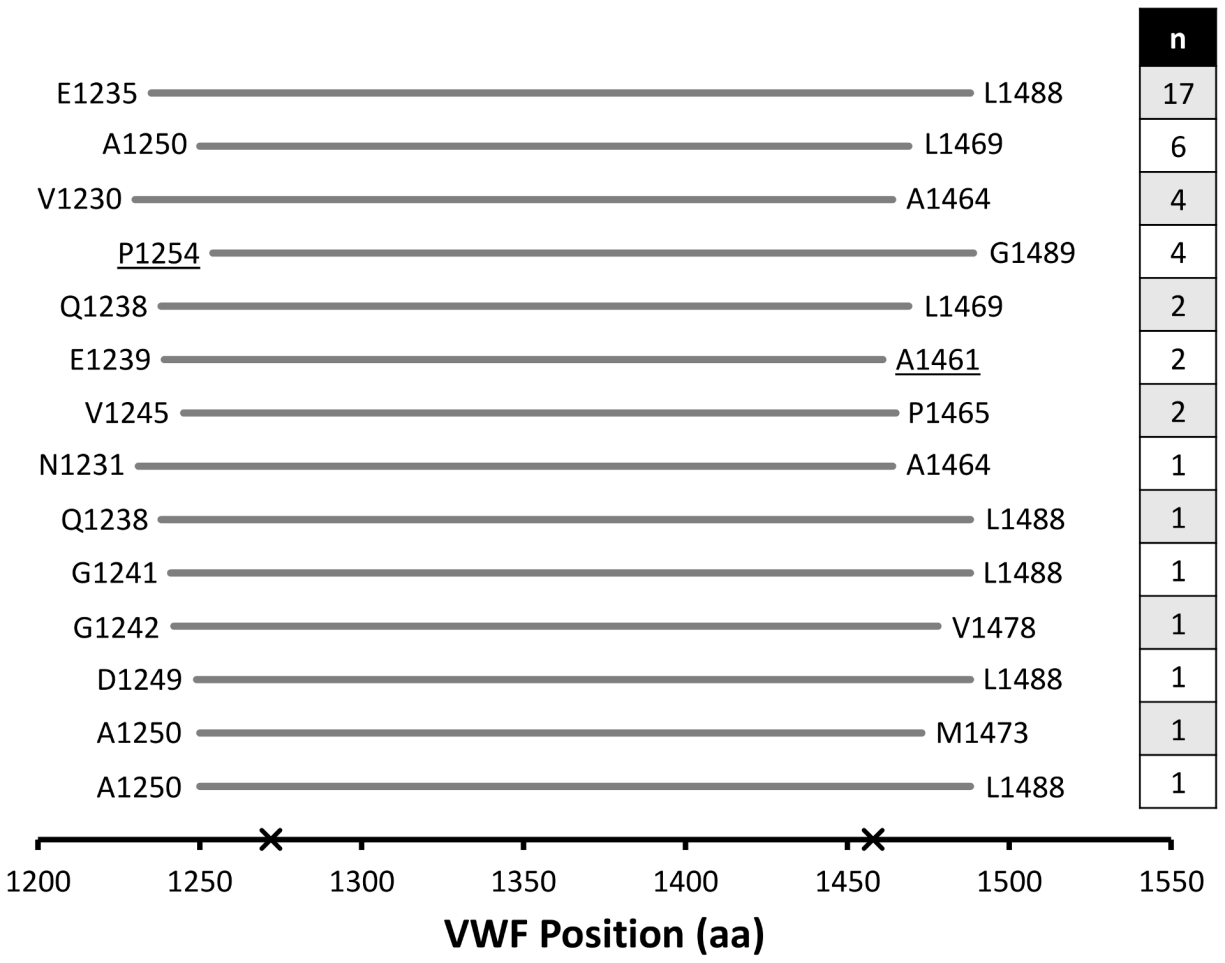
Alignment of platelet-binding VWF fragments. Amino acid segments encoded by VWF cDNA fragment-displaying phage that bound platelets and mapped to the VWF A1 domain. The minimum overlap spans P1254 to A1461 (underlined) and encompasses the two cysteines, C1272 and C1458 (both marked by X), that form the disulfide loop of the A1 domain. The number of times (n) each unique clone was identified is indicated in the right column.

The wtVWF phage library was screened against formalin-fixed platelets, and recovered phage were individually selected at random to confirm platelet binding by ELISA and for subsequent identification by DNA sequencing. Following 3 rounds of panning in the presence or absence of botrocetin, 30% (46/154) and 0% (0/36), respectively, of the selected phage bound to formalin-fixed platelets. None of 48 randomly selected, input (i.e., unscreened) phage exhibited platelet binding in this assay. DNA sequencing revealed that 44 of the 46 ELISA positive phage encoded a VWF fragment in the correct orientation and reading frame for expression as a fusion protein with pIII; the remaining 2 phage did not yield a PCR product and were excluded from further analyses. These 44 phage were composed of 14 unique sequences which all aligned to the A1 domain of VWF (Figure 2) and ranged in length from 254 aa (E1235–L1488) to 220 aa (A1250–L1469), demonstrating that the smaller fragments (∼33 aa–219 aa) within the library have a markedly reduced or absent capacity for platelet binding. Although an antibody to GPIb was used to capture platelets for the confirmatory platelet-binding ELISA, the positive signal for all 44 VWF A1 phage clones demonstrates minimal interference of the capture antibody with VWF A1 phage binding to platelet GPIb. The most over-represented VWF fragment (n = 17) spanned E1235–L1488, suggesting that this particular peptide adopts an optimal structure for GPIb binding or was preferentially expressed or displayed. Alignment of these 14 unique sequences identified a minimum sequence for GPIb binding spanning P1254–A1461, which encompasses the cysteine-cysteine intramolecular loop (C1272–C1458, Figure 2).

Only ∼1∶11,500 phage in the wtVWF library would be expected to display a VWF peptide spanning C1272 to C1458 by chance (see Materials and Methods). The observation that 44 of 154 randomly sampled phage following 3 rounds of platelet panning map to this VWF segment indicates a significant (hypergeometric p<0.0001) enrichment of ∼3,200-fold, corresponding to ∼15-fold enrichment per round of phage selection. These data are consistent with enrichment factors described for other phage display experiments [16].

### Optimal VWF Phage/Platelet Binding Requires the C1272–C1458 Intramolecular Disulfide Bond

**Figure 3:**
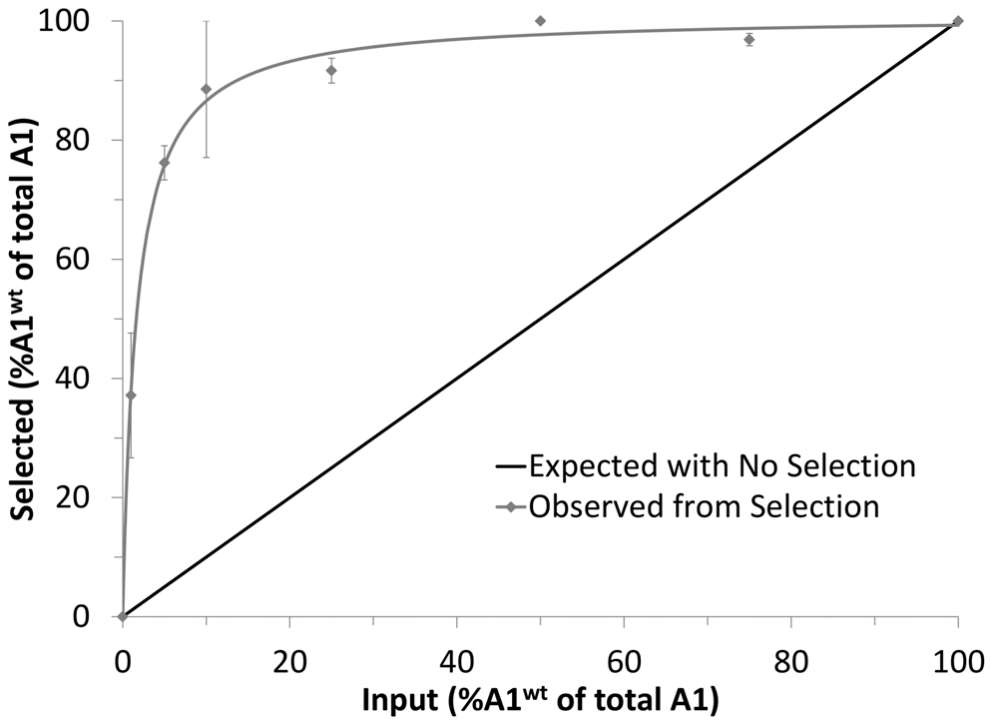
Preferential enrichment of A1^wt^ over A1^C1272/1458A^. A1^wt^ phage were titrated against A1^C1272/1458A^ phage in a mixture composed of 95% A3 phage and 5% total A1 phage (A1^wt^+A1^C1272/1458A^). Following a single round of selection in the presence of botrocetin, randomly picked individual phage clones (48 per condition) were distinguished by RFLP analysis in which A1^wt^ but not A1^C1272/1458A^ was cleavable with PstI. In all conditions, the population of total A1 phage was significantly greater than A3 phage (χ^2^ p-value<0.0001). The observed selected A1^wt^ percent of the total A1 population (mean ± S.D., n = 2) were non-linearly regressed to a 2-site binding model.

The observation that all the phage clones identified above encode a VWF peptide fragment spanning C1272 and C1458 (Figure 2), suggests that the intramolecular disulfide bond known to form between these two cysteines in native VWF [1] is required for optimal platelet binding in this phage display system. To test this hypothesis, a phage clone displaying the VWF A1^wt^ domain from Q1238 to V1476 (A1) was mixed with a derivative of this phage in which both cysteines in the A1 loop had been mutated to alanine (A1^C1272/1458A^). This mixture was then competitively panned against formalin-fixed platelets in the presence or absence of botrocetin with an excess of a control phage displaying the VWF A3 domain (A3). The VWF A3 domain contains an intramolecular disulfide bond similar to that of the A1 domain, and a recombinant VWF A3 fragment has previously been shown not to compete with native VWF for platelet binding [18]. Consistent with this latter report, A1^wt^ phage showed significant enrichment (∼20-fold, χ^2^ p<0.0001) following one round of affinity selection to formalin fixed platelets in the presence of botrocetin and excess A3 phage (Table 1). Similarly, A1^C1272/1458A^ was significantly enriched (12-fold, χ^2^ p<0.0001), though less marked compared to A1^wt^. No enrichment of either phage was seen in the absence of botrocetin. This result demonstrates that formation of the intramolecular disulfide bond within the A1 domain is not absolutely required for platelet binding, consistent with previous reports that a reduced/alkylated VWF A1 fragment can inhibit VWF-mediated platelet aggregation [9], [19]. However, competitive panning between equal amounts of the A1^wt^ and A1^C1272/1458A^ phage against platelets in the presence of botrocetin and excess A3 phage (Table 1) demonstrates minimal enrichment of A1^C1272/1458A^ phage (1.4-fold) compared to wild type A1^wt^ phage (34-fold, χ^2^ p<0.0001). Titration of A1^wt^ against A1^C1272/1458A^ phage in the presence of botrocetin (Figure 3) showed equivalent enrichment of A1^wt^ at an ∼1∶60 A1^wt^:A1^C1272/1458A^ ratio, further highlighting the optimal platelet-binding structure which the C1272–C1458 disulfide bond confers.

**Table 1:** Percent population after single round, competitive panning.

	Input			Without Botrocetin			With Botrocetin			
Condition	A3 (%)	A1^wt^ (%)	A1^C1272/1458A^ (%)	A3 (%)	A1^wt^ or A1^C1272/1458A^ (%)	?^2^ p-value	A3 (%)	A1^wt^ (%)	A1^C1272/1458A^ (%)	?^2^ p-value
**A1** ^wt^ **+A3**	95.0	5.00	0	98.4±1.44	1.65±1.44	0.125	1.39±2.41	98.6±2.41	NA	<0.0001
**A1^C1272/1458A^+A3**	95.0	0	5.00	95.4±2.38	4.61±2.38	0.857	37.0±38.6	NA	63.0±38.6	<0.0001
**A1** ^wt^ **+A1^C1272/1458A^+A3**	95.0	2.50	2.50	97.7±0.42	2.34±0.42	0.223	*11.1±15.8	*85.3±12.8	*3.63±3.25	<0.0001

Values reported (mean ± standard deviation) are averaged from 3 independent screens. Per screen, 38–48 clones randomly selected from each condition were analyzed.

* Identities were determined by DNA sequencing because A1^wt^ and A1^C1272/1458A^ produce the same sized PCR product (884 bp).

**Figure 4:**
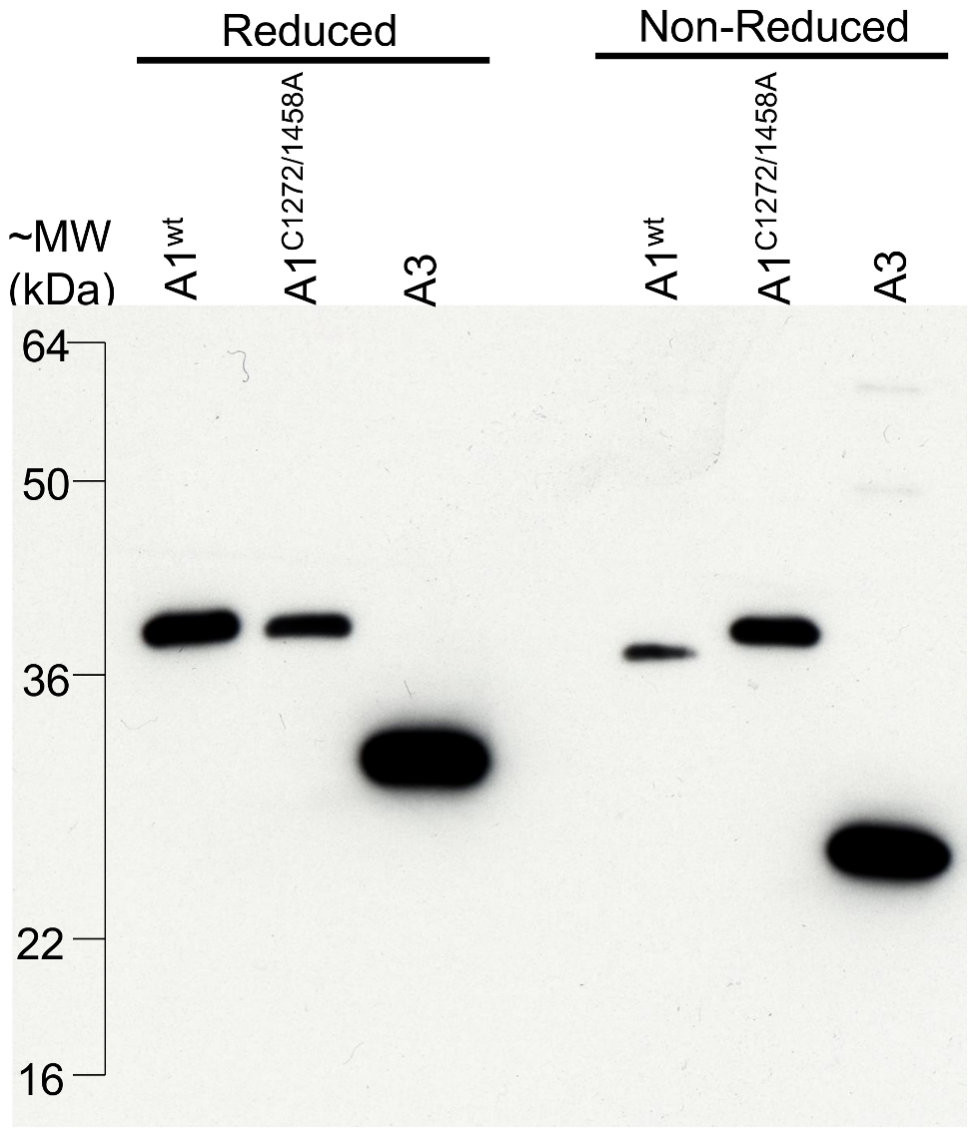
Formation of intramolecular disulfide bonds in *E coli*. Western blot analysis of periplasmic protein under reducing and non-reducing conditions. Intramolecular disulfide bond formation is indicated by a faster migration under non-reducing condition vs. reducing condition. Faint bands (non-reduced) of A3 at ∼45 kDa and ∼60 kDa may be due to intermolecular disulfide bonds.

*E. coli* are known to form disulfide-bonded, recombinant products that reside primarily in inclusion bodies [9], [20]. However, replacement of the gIII signaling sequence with a cotranslational-cotranslocation signaling sequence (TorTss) has been previously shown to improve display of insoluble peptides on filamentous phage [16]. To test whether the modifications to pAYE enabled A1^wt^, A1^C1272/1458A^, and A3 to remain soluble in the periplasm and to form intramolecular disulfide bonds in this oxidative environment, the corresponding phagemids were converted into periplasmic expression plasmids (see Materials and Methods). Western analysis of periplasmic protein harvested by osmotic shock showed an electrophoretic mobility shift for A1^wt^ and A3 (reduced vs. non-reduced) but not for the A1^C1272/1458A^, demonstrating that the cysteine-cysteine loop structure of A1^wt^ and A3 were formed and maintained in the periplasm of *E. coli* (Figure 4). Reduced A1^wt^ exhibited equivalent mobility to A1^C1272/1458A^.

### Mutants of VWF A1 Domain with Enhanced Platelet Binding

To explore the spectrum of potential amino acid substitutions within the VWF A1 domain on platelet binding, a randomly mutagenized phage display library was constructed by error-prone PCR. This library (mtA1) contained ∼2×10^6^ independent phage clones, sufficient to cover all 4,420 single amino acid substitutions and ∼20% of 9,724,000 double substitutions. Sequence analysis of 16 randomly selected clones identified 2–6 nucleotide substitutions per clone. To select for specific amino acid substitutions with enhanced platelet binding, the mtA1 phage library was mixed with the wtVWF fragment library at a ratio of 1∶100 and competitively screened for binding to formalin-fixed platelets. Both the wtVWF and the mtA1 libraries contained wild type fragments of the VWF A1 domain against which mutants compete. The input library (5×10^10^ pfu) was composed of 99% wtVWF library (4.95×10^10^ pfu, representing ∼7.7×10^6^ individual clones) +1% mtA1 library (5×10^8^ pfu, representing ∼2×10^6^ individual clones). Of the ∼7.7×10^6^ independent clones in the wtVWF library, ∼670 independent clones represented the wild type A1 domain (see Methods and Materials) at ∼6,400-fold coverage (4.95×10^10^/7.7×10^6^). Given the input composition, each independent mutant from the mtA1 library was represented ∼250-fold. Taken together, we estimated that each independent mutant is competing against an ∼17,000-fold excess (∼6,400×670/∼250) wild type A1 fragments. After 4 rounds of affinity selection, recovered phage were analyzed by ELISA and DNA sequencing. Seventy-two ELISA positive phage (43 from the +botrocetin selection and 29 −botrocetin) were analyzed by DNA sequencing. All 72 aligned to the VWF A1 domain, and only 2 of the 72 contained wild-type sequence. Of the 70 mutant phage, 68 carried mutations resulting in at least 1 amino acid substitution (Table 2). Affinity maturation through multiple rounds of panning reduced the complexity of recovered phage as observed by the decrease in the number of independent clones from 25 (round 3, data not shown) to 10 (round 4). Only 2 amino acid substitutions (S1370G and I1372V) were identified independently in both the presence and absence of botrocetin. These were also the two most highly represented mutations, with I1372V ∼50% of the phage isolates under both conditions and S1370G at ∼10–20%.

**Table 2:** A1 mutations selected after 4 rounds of panning.

Without Botrocetin		With Botrocetin	
Mutation	Count (n)	Mutation	Count (n)
L1296Q/K1348/S1378A/Q1449L	2	L1257P	2
D1302G/I1309V	1	L1257P/D1261G	4
M1303V/F1366I/I1410T/I1412V	1	S1273G	1
S1310Y	1	R1287G/F1397L	1
A1317(Silent mutation)/S1370G	1	S1370G	9
S1345T	1	I1372V	22
Y1363H/I1372N/L1446P	1	V1401L	1
S1370G	2	I1452V	1
I1372V	13	Y1456T	1
I1380V/K1423E/E1445G	1	WT (silentmutation)	1
I1412V	1		
N1421S/Q1448L	1		
WT	2		
WT (silent mutation)	1		

## Discussion

The interaction between VWF and GPIb on the platelet membrane surface is a critical early step in hemostasis. Adherence of native, circulating VWF to platelet GPIb requires a conformational change that exposes the VWF A1 domain. We demonstrate that VWF fragments displayed on the surface of filamentous phage can bind platelets, and use this system to interrogate the full VWF sequence for platelet binding function. Our results confirm that the A1 domain is primarily responsible for VWF-platelet interaction and demonstrate that this unbiased approach fails to detect an interaction of any other region of VWF with formalin fixed platelets. We also show that formation of the C1272–C1458 disulfide bond is required for optimal platelet binding, though residual binding is maintained in the absence of this structure. By coupling random mutagenesis with phage display, we identify additional residues modulating the VWF-platelet interaction, including gain-of-function mutations within the VWF A1 domain.

The architecture of the VWF A1 domain and adjacent structures is critical to regulating the VWF-platelet interaction. Mapping VWF residues involved in GPIb interaction by filamentous phage relies on faithful emulation of native protein structures by recombinant proteins expressed in *E. coli.* In previous reports, bacterially expressed VWF A1 peptide purified from inclusion bodies required renaturation/refolding for functional competency [8], [9], [20]. Our results demonstrate that directing VWF peptide fragments to the periplasm, either by fusion to the phage pIII coat protein or by addition of a cotranslational-translocation signaling sequence, facilitates solubility and formation of the physiological disulfide bond within the A1 domain (Figures 2, 3, 4 and Table 2). Though not required for binding, the C1272–C1458 intramolecular disulfide bond optimizes the structure of the VWF A1 domain for binding to platelet GPIb [18]. Our results also confirm previous reports demonstrating that glycosylation of VWF residues surrounding the A1 domain is not required for platelet binding [8], [21]. The minimal overlapping VWF sequence (P1254–A1461, Figure 2) found in this study suggests that residues N-terminal to the A1 domain may participate in the VWF-platelet interaction whereas C-terminal flanking residues may be less important. Enrichment of the most over-represented fragment (E1235–L1488, Figure 2) for platelet binding contrasts with the previously reported inhibitory effect of Q1238–E1260 on platelet binding by a VWF fragment recombinantly expressed from mammalian cells [11]. Taken together, these results suggest that glycosylation of N-terminal flanking residues within the Q1238–E1260 segment may hinder VWF-GPIb interaction. However, we cannot exclude the possibility that the longer N-termini may also contribute to improved phage solubility/recovery/production during the additional rounds of panning required for signal amplification. Although previously reported recombinant VWF A1 fragments spontaneously bind platelets [20], [22], no binding of A1 phage to platelets was detected in the absence of botrocetin (Table 1). The latter observation could result from the low effective concentrations of the A1 fragment in the phage context (<1 nM) or due to conformational changes resulting from the phage fusion protein.

**Figure 5:**
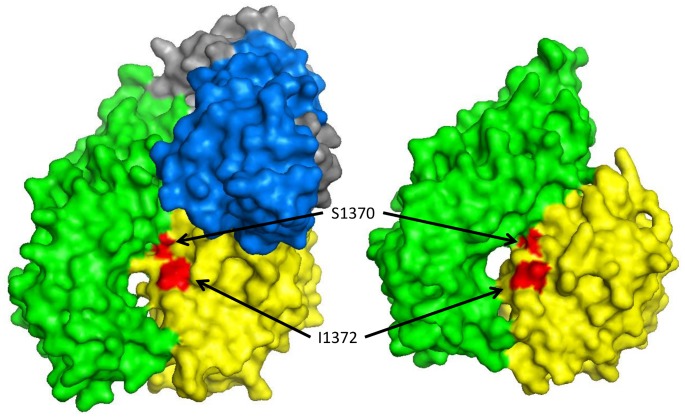
Location of A1 mutations (S1370G and I1372V) found to enhance platelet binding in the presence and absence of botrocetin. In space filled crystal structures of both the ternary A1-GPIb-botrocetin ternary complex (PDB 1U0N, left) and the binary A1-GPIb binary complex (PDB 1SQ0, right) visualized in PyMOL, S1370 (red) of the A1 domain (yellow) directly contacts GPIb (green) whereas I1372 (red), though also located at the A1-GPIb interface, does not [2], [4]. The botrocetin heterodimer (blue and gray) is shown as part of the ternary complex (left).

In type 2B VWD, mutation(s) that enhance the affinity of the A1 domain for GPIb (cataloged at www.vwf.group.shef.ac.uk/index.html) result in spontaneous VWF-platelet binding, causing loss of high molecular weight VWF multimers and thromobocytopenia due to clearance of VWF-platelet complexes. Most type 2B VWD mutations lie within the VWF A1 domain and are thought to destabilize interactions within the A1 domain that regulate its tertiary structure inhibiting formation of the interface with GPIb [2], [23]. S1370G and I1372V lie in the α3β4 loop of the A1 domain, a component of both ∼1,700 Å^2^ and ∼900 Å^2^ noncontiguous surfaces that contact GPIb (Figure 5), and may directly enhance the affinity of the interface [2]. The similar frequency of I1372V in the presence and absence of botrocetin suggest that the latter cofactor does not markedly enhance the affinity of this mutant A1 domain for GPIb. Interestingly, whereas S1370G enhance platelet binding, S1370R has previously been reported to block binding of recombinant A1 to platelets by either ristocetin or hydrodynamic forces [24], implicating a critical role for S1370 in regulating VWF-GPIb binding. Characterization of a larger set of mutants by phage display may further define the contributions of each amino acid of the A1 domain to platelet binding.

Preventing platelet adhesion and subsequent aggregation at sites of vascular injury by blocking the VWF/GPIb interaction represent an attractive target for the treatment of important human diseases including thrombotic thrombocytopenic purpura, acute coronary syndrome, and stroke. Potential therapeutic approaches include antibodies against GPIb [25] or VWF [26], recombinant VWF fragments [9], [27], and DNA aptamers [22], [28]. The phage display approach described here may identify novel VWF-related peptides with potential for applications as novel antithrombotic therapeutics.

## Supporting Information

Table S1 **Phagemid features.**
(DOCX)

Text S1 **Sequence of pAYE.** The sequence of pAYE with the cDNA encoding the A1 domain of wild type VWF (Q1238–V1476) inserted.
(DOCX)
