# Supplementary material for: Functional Display of Platelet-Binding VWF Fragments on Filamentous Bacteriophage
Source: PLoS One. 2013 Sep 3;8(9):e73518. doi: 10.1371/journal.pone.0073518 (PMC3760814; doi:10.1371/journal.pone.0073518)
Supplement: Table S1 — Phagemid features. (DOCX) [file pone.0073518.s001.docx]

# Table S1

| **Feature** | **Phagemid Position (bp)** |
| --- | --- |
| Ampicillin resistance | 201-1061 |
| ColE1 origin of replication | 1216-1835 |
| *lac* promotor | 2144-2200 |
| HindIII restriction site | 2236 |
| TorT signaling sequence | 2269-2322 |
| Amber stop (*glnV* or *supE*) | 2326-2328 |
| SfiI restriction site | 2330 |
| AscI restriction sites | 2337 and 3076 |
| VWF A1 domain | 2350-3066 |
| E tag | 3091-3129 |
| NotI restriction site | 3084 |
| BamHI restriction site | 3112 |
| Gly-ser rich linker (G2) of gIII | 3139-3270 |
| CT domain of gIII | 3271-3720 |
| EcoRI restriction site | 3728 |
| M13 origin of replication | 3944-4417 |
